# Sarcopenia Prevalence and Risk Factors Among Older Adults in Bangkok, Thailand: A Cross-Sectional Study

**DOI:** 10.7759/cureus.63483

**Published:** 2024-06-29

**Authors:** Kasidid Lawongsa, Jitrawee Tepakorn

**Affiliations:** 1 Family Medicine, Phramongkutklao Hospital, Bangkok, THA

**Keywords:** prevalence, sarcopenia, public health, risk factors, aging population

## Abstract

Objectives: This study aimed to investigate the prevalence of sarcopenia among older Thai adults residing in Bangkok and identify associated risk factors.

Methods: This cross-sectional study included Thai adults aged ≥60 years. All participants underwent assessments using the Yubi-wakka test, anthropometric measurements, bioelectrical impedance analysis, handgrip strength tests, and physical performance evaluations. Information on demographic characteristics, chronic health conditions, nutritional status, and medication use was also collected. Sarcopenia was diagnosed according to the 2019 Asian Working Group for Sarcopenia criteria. Statistical analyses were performed using the independent t-test, Chi-square test, and logistic regression to identify risk factors.

Results: Among the 600 participants, the overall prevalence of sarcopenia was 19%. The multivariate analysis identified 13 significant risk factors associated with sarcopenia, including age ≥75 years (odds ratio [OR]=10.6, 95% confidence interval [CI]=3.7-30.2), higher education level (year) (OR=0.85, 95% CI=0.74-0.98), lower income level (OR=5.4, 95% CI=1.4-21.4), Barthel index <90 (OR=11.0, 95% CI=3.5-34.5), lower body mass index (OR=0.7, 95% CI=0.6-0.8), low calf circumference (OR=7.6, 95% CI=2.5-23.3), fall in the past year (OR, 3.1; 95% CI, 1.4-6.6), frailty (OR, 2.3; 95% CI, 1.1-4.5), malnutrition (OR=3.5, 95% CI=1.3-9.3), history of stroke (OR=7.5, 95% CI=1.3-41.4), vitamin D deficiency (OR=9.4, 95% CI=1.1-82.5), knee osteoarthritis (OR=6.3, 95% CI=1.57-25.31), and malignancy (OR=4.8, 95% CI=1.01-22.70).

Conclusion: This study evaluated the sarcopenia status across a diverse demographic of older Thai adults using comprehensive assessments, and examined the impact of socioeconomic factors and various chronic conditions on the occurrence of sarcopenia.

## Introduction

In Thailand, as in many other nations, the shift toward an older demographic emphasizes the necessity of comprehending sarcopenia prevalence and associated risk factors to effectively address its public health impact [1,2]. Although research on sarcopenia prevalence in Thailand is limited, emerging evidence suggests a growing trend [3], aligning with global patterns. With Thailand experiencing a rapidly aging population and shifts in lifestyle and dietary habits, unique challenges arise in addressing sarcopenia and its related health implications.

According to the 2019 consensus of the Asian Working Group for Sarcopenia (AWGS), sarcopenia is defined by both low muscle mass and impaired muscle function, encompassing an individual’s strength and physical performance [4,5] which significantly impacts the elderly overall health and well-being [6-8]. In Thailand, the prevalence of sarcopenia among community-dwelling individuals aged ≥60 years was 9.6% in men and 20.4% in women [9]. Another study found rates of 11.1% in men and 20.5% in women [10]. In Japan, prevalence among those aged >65 years was 21.8% in men and 22.1% in women [11]. In China, rates among those aged >60 years were 11.3% in men and 9.8% in women [12]. In Taiwan, rates among older adults were 9.3% in men and 4.1% in women. In Hong Kong, prevalence among older men was 9.4% [8]. These statistics may vary across studies and populations.

Timely detection of sarcopenia and implementation of various intervention strategies to enhance muscle performance are essential for mitigating its adverse effects. Furthermore, identifying modifiable risk factors is crucial for developing targeted interventions to reduce the disease burden. Lifestyle elements, such as physical inactivity, inadequate nutrition, and comorbidities like diabetes and obesity, likely contribute to sarcopenia. Additionally, socioeconomic factors, cultural influences, and healthcare accessibility may significantly influence sarcopenia risk profiles in Thai populations [10].

This study aimed to comprehensively investigate sarcopenia prevalence and associated risk factors among Thai adults considering demographic, lifestyle, and health-related factors. Findings from this research will inform the development of tailored interventions and public health policies aimed at reducing the sarcopenia burden and enhancing the health and well-being of aging Thai individuals.

## Materials and methods

Study design and population

This cross-sectional study included Thai adults aged ≥60 years residing in Bangkok, Thailand, who were capable of effective communication. The sample size for the study was estimated to ensure reliable prevalence estimates and adequate power for identifying risk factors for sarcopenia. Based on previous studies [13], an adjustment factor was applied to account for potential non-response and dropouts. We estimated a 30% non-response rate. Therefore, the adjusted sample size was calculated as 530 participants, the sample size was increased to 600 participants, ensuring sufficient data quality and robustness for analyzing sarcopenia prevalence and associated risk factors​ Individuals who were physically and/or mentally incapable of performing the required assessments, such as those who were bedridden, at an end-of-life stage, with severe dementia, or unable to walk independently, were excluded. Individuals with contraindications for bioelectrical impedance analysis (BIA) were also excluded. Initially, 605 participants were considered for the study. However, five participants were excluded due to physical incapability (one bedridden, three unable to walk independently), and one was excluded due to contraindications for BIA (pacemaker or implanted electronic device). Therefore, 600 participants were included in the final analysis.

Ethical approval for the study was obtained from the Ethics Committee of the Institutional Review Board of the Royal Thai Army Medical Department (IRBTA1468/2565). All data used in the analysis were anonymized and referenced solely by ID numbers to protect participants’ confidentiality. Personal identifiers and sensitive information were omitted to ensure the privacy of participants.

Assessments

All participants were assessed by experienced physical therapists from the research center of the hospital.

Anthropometric Measurements

The assessed anthropometric measurements included standing height, weight, body mass index (BMI), Yubi-wakka (finger-ring) test, and calf circumference. Calf circumference was measured while participants were in a seated position using a measuring tape (Abbot Laboratories, Irving, TX, USA). All measurements were conducted by qualified personnel between 9:00 a.m. and 3:00 p.m. on the same day.

Assessment of Sarcopenia Using the AWGS Criteria

Sarcopenia diagnosis was based on the 2019 AWGS criteria, encompassing decline in muscle strength, physical performance, and muscle mass. Specifically, sarcopenia diagnostic criteria included muscle mass below 7.0 kg/m2 for men and 5.7 kg/m2 for women, grip strength below 28 kg for men and 18 kg for women, and/or inability to complete a five-time chair stand within 12 seconds [5].

Muscle Mass and Body Composition

Appendicular skeletal muscle mass (ASM) and body fat mass were assessed using BIA (InBody720; Biospace, Seoul, South Korea). Participants were instructed to fast for 12 hours and refrain from drinking water for four hours before the measurements. Coffee and alcohol were prohibited the day before the measurements [14]. Participants stood barefoot on the machine, ensuring contact with both the front and back electrodes. The ASM index (ASMI) was calculated by summing the muscle mass of all four limbs and adjusting it using the squared height (ASM/height [kg/m2]) [15].

Muscle Strength

Handgrip strength of the dominant hand was measured using a Smedley hand dynamometer (TKK-5401 Digital Grip Force Meter, Grip D; Takei Equipment Industries, Saga, Japan). Two measurements were obtained, and the higher value was selected for analysis [8].

Physical Performance

Physical performance was assessed using the five-time chair stand test, which measures the time taken by participants to transition from a seated position, with arms folded across their chest, to a standing position for five repetitions. The duration of each participant’s chair stand was recorded in seconds. Participants who were unable to perform the chair stand test were classified as having poor physical ability [16]. Those who took more than 12 seconds to complete the test were classified as having reduced physical performance.

Data collection

Data collection was conducted through questionnaires. Demographic and socioeconomic data included age, sex, educational background (years), income, and living arrangements. Income was categorized into two levels: <USD 135 and ≥USD 135 (equivalent to <THB 5,000 and ≥THB 5,000, respectively; 1 USD = 37 THB). This categorization was based on the median income level of the study population, ensuring a balanced distribution between lower and higher income groups. Additionally, the threshold of THB 5,000 per month is commonly used in Thailand as a benchmark to distinguish between lower-income individuals [17], who may face more significant socioeconomic challenges, and those with relatively better financial stability. This division helps in identifying the potential impact of socioeconomic status on sarcopenia prevalence and associated risk factors.

Health-related data included chronic conditions and medication use. We considered the following chronic conditions: fall in the past year, frailty (assessed using the Frailty Index, participants with a total score of 3 or more were classified as frail) [18], hypertension, diabetes mellitus, dyslipidemia, osteoporosis, malignant neoplasms, chronic renal failure, low vitamin D levels, gout, heart disease, respiratory diseases (including asthma and chronic obstructive pulmonary disease), gastrointestinal diseases (including gastroesophageal reflux disease and dyspepsia), Parkinson’s disease, stroke, dementia, rheumatoid arthritis, and knee osteoarthritis. Medication reviews were conducted during both community and hospital visits. Physicians examined all medication containers, including prescriptions and over-the-counter medications used in the previous month. Hospital medication records were verified using the electronic medical record system.

Sarcopenia Risk Factors

To identify risk factors for sarcopenia, in addition to the above, we evaluated the participants’ nutritional status and activities of daily living. Nutritional status was assessed based on the Malnutrition Screening Tool (MST) and Mini Nutritional Assessment short-form (MNA-SF) scores. The ability to perform activities of daily living was assessed using the Barthel index.

The MST [19] is a quick and simple screening method used to identify adults at risk for malnutrition, based on two questions about recent unintentional weight loss and changes in appetite. A score of 0-1 points indicates a low risk for malnutrition, while a score of 2 or more points signifies a high risk for malnutrition.

The MNA-SF [19] categorizes participants’ nutritional status as follows: a score of 0-7 points indicates malnutrition, requiring comprehensive nutritional assessment and immediate intervention; a score of 8-11 points suggests a risk for malnutrition, necessitating further evaluation, monitoring, and nutritional intervention to prevent progression; and a score of 12-14 points indicates normal nutritional status.

Additionally, mobility performance, muscle strength, and musculoskeletal examinations were conducted to assess muscle strength and power, with a focus on proximal muscles.

Statistical Analysis

For analysis purposes, participants were divided into sarcopenia and non-sarcopenia groups. Continuous variables were summarized as median ± standard deviation, while categorical variables as numbers and percentages.

Differences in continuous and categorical variables between the two groups were evaluated using the independent t-test and Chi-square test, respectively. Prior to conducting the t-test, data were assessed for normality and homogeneity of variances. Normality was evaluated using the Shapiro-Wilk test, and homogeneity of variances was assessed using Levene's test. Only data that satisfied these assumptions were analyzed using the t-test. In cases where the assumptions were not met, appropriate non-parametric tests, such as the Mann-Whitney U test, were employed to ensure the validity of the statistical analysis.

Multivariable logistic regression was employed to identify factors associated with sarcopenia. This approach allowed for the adjustment of potential confounding variables. Multivariable models were constructed by including covariates with a p-value < 0.05 in univariate models, using a stepwise backward elimination approach. This method enabled the identification of independent risk factors while controlling for the influence of other variables.

## Results

Sarcopenia prevalence

**Figure 1:**
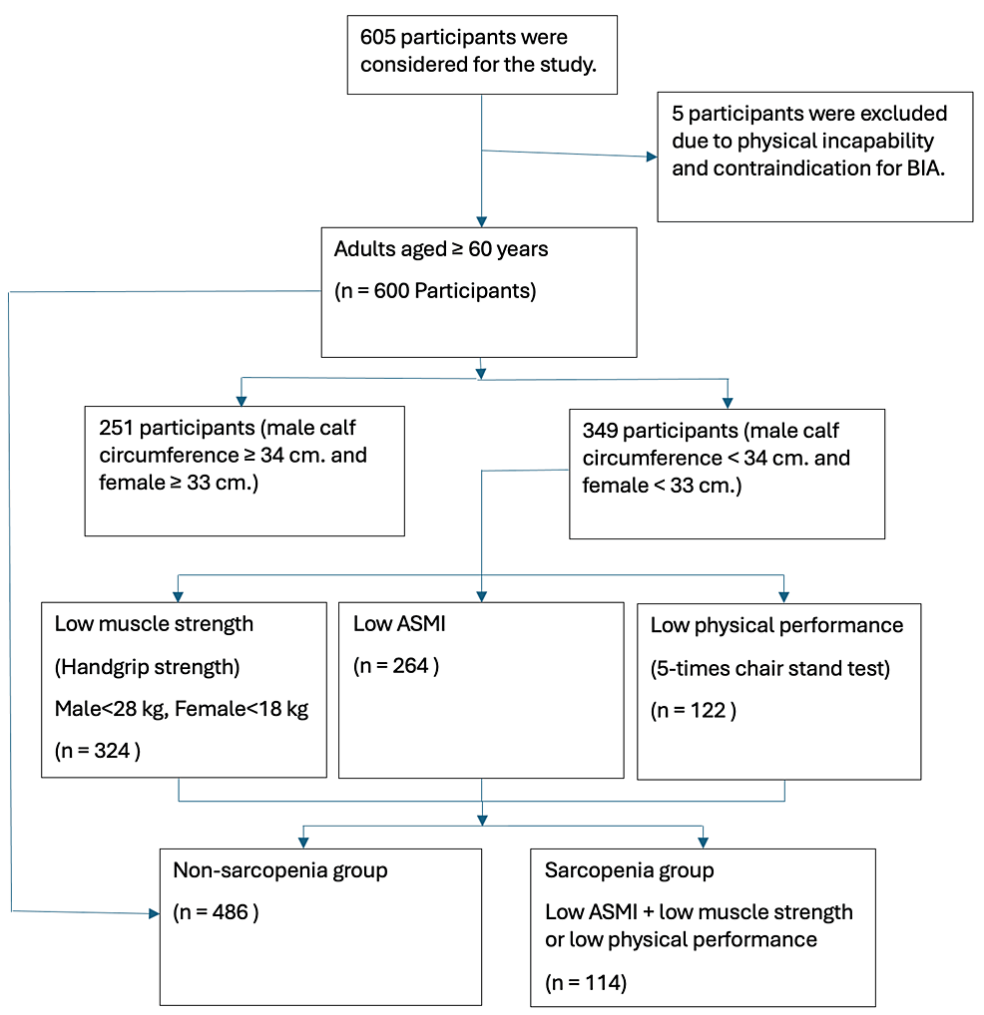
Flowchart of study enrollment and sarcopenia diagnosis. BIA: bioelectrical impedance analysis, ASMI: appendicular skeletal muscle mass index

Out of the 600 participants enrolled in the study, 349 (58.2%) were found to have a calf circumference of less than 34 cm for males and less than 33 cm for females after screening for sarcopenia. Utilizing BIA, hand grip strength assessments, and physical performance tests, 114 (19%) participants were identified as having sarcopenia (Figure 1).

Participants' characteristics

As shown in Table 1, the average age of the study population was 70 years (P < 0.001). The prevalence of sarcopenia was higher among females (86 (75.4%)).

**Table 1:** Baseline characteristics of sarcopenia group. ^a^ p-value < 0.05 was considered statistically significant. Values are shown as mean±standard or number of participants (%). BMI: body mass index, ASMI: appendicular skeletal mass index, Low calf circumference: Men < 34 cm, Women < 33 cm, MST: malnutrition screening tool, MNA: mini nutritional assessment.

Variables (units)		Overall	Non-sarcopenia	sarcopenia	p-value^a^
No. of participant			486(81%)	114(19%)	-
Sex	Female	392(65.3%)	306(63%)	86(75.4%)	0.022^*^
Basic attributes					
Age	-	70±7	68±6	75±7	<0.001^*^
	≥75	142(23.7%)	82(16.9%)	60(52.6%)	<0.001^*^
Years of education (year)		10.33±4.07	10.77±4.16	8.54±3.1	<0.001^*^
Income	<135USD	348(59.8%)	294(62.8%)	54(47.4%)	0.033^*^
Barthel index (<90)		160(26.7%)	102(21%)	58(50.9%)	<0.001^*^
Living arrangement	(alone)	210(36.1%)	288(61.5%)	84(73.7%)	0.087
Alcohol (≥3 units/day)		32(5.3%)	18(3.7%)	14(12.3%)	0.009^*^
Smoking		22(3.7%)	12(2.5%)	10(8.8%)	0.023^*^
Yubi-wakka	Bigger	330(55%)	306(63%)	24(21.1%)	<0.001^*^
	Just-fit	120(20%)	84(17.3%)	36(31.6%)	-
	Smaller	150(25%)	96(19.8%)	54(47.4%)	-
Fall in the past year		54(9%)	36(7.4%)	18(15.8%)	0.005^*^
Frailty (frail scale ≥ 3)		48(8%)	28(5.8%)	20(17.5%)	<0.001^*^
Anthropometric measurements					
Weight (kg)		60.5±12.3	63±11.3	50.1±10.7	<0.001^*^
Height (cm)		158±8	159±8	153±7	<0.001^*^
BMI (kg/m2)	-	24.32±4.58	25.04±4.43	21.34±3.94	<0.001^*^
	<18.5	54(9%)	42(8.6%)	12(10.5%)	0.527
Handgrip strength	-	22.83±7.72	24.16±7.78	17.15±3.98	<0.001^*^
	Low	324(54%)	222(45.7%)	102(89.5%)	<0.001^*^
5-times chair stand	-	10.11±3.36	9.84±3.45	11.23±2.73	<0.001^*^
	≥12sec	122(20.3%)	66(13.6%)	56(49.1%)	<0.001^*^
Calf circumference		36.32±3.86	37.02±3.89	33.32±1.65	<0.001^*^
ASMI (kg/m2)		6.41±1.03	6.65±0.94	5.45±0.76	<0.001^*^
MST score	(≥2)	174(29%)	124(25.5%)	50(43.9%)	0.006^*^
MNA score	(≤11)	192(32.7%)	126(26.6%)	66(57.9%)	<0.001^*^
Malnutrition		176(29.3%)	112(23%)	64(56.1%)	<0.001^*^
Vitamin D deficiency		34(5.7%)	12(2.5%)	22(19.3%)	<0.001^*^
Malignancy		50(8.3%)	24(4.9%)	26(22.8%)	<0.001^*^
Knee osteoarthritis		66(11%)	42(8.6%)	24(21.1%)	0.007^*^

Compared with the non-sarcopenia group, the sarcopenia group had significantly lower average BMI (21.3 ± 4.0 vs. 25.0 ± 4.4, P < 0.001), lower average handgrip strength (17.2 ± 4.0 vs. 24.2 ± 7.8, P < 0.001), longer average five-time chair stand duration (11.2 ± 2.8 vs. 9.8 ± 3.5, P < 0.001), and low calf circumference (33.3±1.7 vs. 37.0±3.9, P < 0.001). The average ASMI was lower in the sarcopenia than in the non-sarcopenia group (5.5 ± 0.8 vs. 6.65 ± 0.9, P < 0.001). According to the Yubi-wakka test results, the prevalence of sarcopenia was 54 (47.4%) in the smaller group and 36 (31.6%) in the just-fit group.

In terms of socioeconomic factors, participants in the sarcopenia group had fewer years of education than those in the non-sarcopenia group (8.5 ± 3.1 vs. 10.8 ± 4.2, P < 0.001). The prevalence of sarcopenia was 54 (47.4%) among those with an income of <USD 135. The prevalence of sarcopenia among those living alone, fall in the past year, frailty, alcohol drinkers (≥3 units/day), and smokers was 84 (73.7%), 18 (15.8%), 20 (17.5%), 14 (12.3%), and 10 (8.8%), respectively.

In terms of nutritional status, the proportion of participants with an MNA-SF score of <12 was significantly higher in the sarcopenia than in the non-sarcopenia group (66 (57.9%) vs. 126 (26.6%), P < 0.001). Additionally, a significantly higher number of participants in the sarcopenia group had an MST score of ≥2 compared with those in the non-sarcopenia group (50 (43.9%) vs. 124 (25.5%), P = 0.006).

In terms of chronic conditions, compared with those in the non-sarcopenia group, participants diagnosed with sarcopenia had a higher prevalence of malnutrition (64 (56.1%)), vitamin D deficiency (22 (19.3%)), malignancy (26 (22.8%)), and knee osteoarthritis (24 (21.1%)).

Sarcopenia risk factors

The results of the multivariate analysis identified the following significant risk factors for sarcopenia (Table 2): age (≥75 years; adjusted odds ratio [aOR], 10.60; 95% confidence interval [CI], 3.72-30.17), years of education (aOR, 0.85; 95% CI, 0.74-0.98), income of <USD 135 (aOR, 5.44; 95% CI, 1.38-21.40), Barthel index of <90 (aOR, 10.96; 95% CI, 3.48-34.51), BMI (aOR, 0.70; 95% CI, 0.6-0.81), low calf circumference (aOR, 7.63; 95% CI, 2.50-23.34), fall in the past year (aOR, 3.07; 95% CI, 1.43-6.61), frailty (aOR, 2.25; 95% CI, 1.13-4.47), malnutrition (aOR, 3.45; 95% CI, 1.28-9.29), stroke (aOR, 7.46; 95% CI, 1.34-41.40), vitamin D deficiency (aOR, 9.43; 95% CI, 1.08-82.51), knee osteoarthritis (aOR, 6.31; 95% CI, 1.57-25.31), and malignancy (aOR, 4.78; 95% CI, 1.01-22.70).

**Table 2:** Univariate and multivariate analysis for risk factors of sarcopenia. ^a^ Multivariate models adjusting for covariates with p-value<0.05 in univariate models with stepwise backward logistic regression. ^b^ p-values<0.05 were considered statistically significant. BMI: body mass index, Low calf circumference: Men < 34 cm, Women < 33 cm

Variables (units)		Crude OR (95%CI)	p-value^b^	Adjusted OR^a^ (95%CI)	p-value^b^
Age	<75	Ref.			
	≥75	5.474(2.95-10.17)	<0.001^*^	10.60(3.72-30.17)	<0.001^*^
Years of education (year)		0.86(0.79-0.93)	<0.001^*^	0.85(0.74-0.98)	0.031^*^
Income	≥135USD	Ref.			
	<135USD	1.877(1.05-3.37)	0.034^*^	5.44(1.38-21.40)	0.015^*^
Barthel index	≥90	Ref.			
	<90	3.90(2.13-7.13)	<0.001^*^	10.96(3.48-34.51)	<0.001^*^
BMI		0.77(0.70-0.85)	<0.001^*^	0.70(0.6-0.81)	<0.001^*^
Calf circumference	Normal	Ref.			
	Low	3.35(2.04-5.49)	<0.001^*^	7.63(2.50-23.34)	<0.001^*^
Fall in the past year		2.34(1.28-4.30)	0.006^*^	3.07(1.43-6.61)	0.004^*^
Frailty	Frail scale <3	Ref.			
	Frail scale ≥3	3.48(1.88-6.44)	<0.001^*^	2.25(1.13-4.47)	0.021^*^
Malnutrition	No	Ref.			
	Yes	3.39(1.87-6.15)	<0.001^*^	3.45(1.28-9.29)	0.014^*^
Vitamin D deficiency	No	Ref.			
	Yes	4.88(1.84-12.92)	0.001^*^	9.43(1.08-82.51)	0.043^*^
Knee osteoarthritis	No	Ref.			
	Yes	2.82(1.29-6.14)	0.009^*^	6.31(1.57-25.31)	0.009^*^
Malignancy	No	Ref.			
	Yes	5.13(2.17-12.15)	<0.001^*^	4.78(1.01-22.70)	0.049^*^

## Discussion

In the present study, the prevalence of sarcopenia among older adults in urban Thailand, based on the 2019 AWGS criteria, was 19%, similar to a previous study's 17% [9]. Among the evaluated factors, including demographics, nutritional status, physical activity, and chronic health conditions, age ≥75 years, fewer years of education, lower income, Barthel index of <90, low BMI, low calf circumference, falling, frailty, malnutrition, stroke, vitamin D deficiency, knee osteoarthritis, and malignancy were identified as risk factors for sarcopenia in this population.

Sarcopenia prevalence

In our study, 19% of older adults living in urban areas of Bangkok, Thailand, were diagnosed with sarcopenia based on the 2019 AWGS criteria. This significant prevalence suggests that nearly one in five older individuals in this group is affected by sarcopenia. The higher rate found in females (21.9%), calculated as the number of women with sarcopenia divided by the total number of women, compared with males (13.5%), calculated in the same manner indicates a sex disparity consistent with global patterns, where women are more likely to develop sarcopenia, possibly due to factors such as lower baseline muscle mass, hormonal changes during menopause, and higher prevalence of conditions like osteoporosis that exacerbate muscle loss. These factors, combined with age-related declines in muscle mass and strength, contribute to the higher rates of sarcopenia observed in females. The prevalence of sarcopenia observed in this study was similar to that reported by Kim et al. [20] in a study of community-dwelling adults aged ≥70 in Korea (males, 20.1%; females, 29.2%). Both found higher rates in females compared to males. Although the proportions differ, they highlight the consistent trend of higher sarcopenia prevalence in females.

Sarcopenia risk factors

The high prevalence of sarcopenia found in this study is likely due to several contributing factors. Thailand’s rapidly aging population faces physiological changes linked to aging, such as hormonal shifts, reduced physical activity, and nutritional deficiencies, all of which promote sarcopenia, as shown in this study and corroborated by prior research [10,21]. Poor nutritional status, indicated by lower MNA-SF scores and higher MST scores, leads to muscle loss from malnutrition and insufficient protein intake. Additionally, low physical activity levels, common among older adults, significantly impact muscle mass and strength. Chronic conditions like diabetes, hypertension, and cardiovascular diseases exacerbate muscle degradation through chronic inflammation and metabolic issues. Socioeconomic factors, such as lower income and education levels, further contribute by limiting access to healthcare, nutritious food, and physical activity opportunities, thereby increasing the risk for sarcopenia [19].

We identified lower education and income levels as risk factors for sarcopenia. According to Lochlainn et al. [22], both educational attainment and income levels significantly affect the risk of developing sarcopenia. Lower educational levels often lead to reduced health literacy, which impacts individuals’ understanding of nutritional needs and the importance of physical activity. This lack of knowledge can result in poorer health behaviors, such as inadequate diet and insufficient exercise, which are essential for maintaining muscle mass and strength in older adults. Similarly, lower income is closely linked to an increased risk for sarcopenia, as financial constraints can limit access to nutritious food, healthcare services, and opportunities for physical activity.

It is important to note that lower income can be both a cause and a consequence of lower education. Individuals with lower education levels may have fewer economic opportunities, resulting in lower income, which further limits their ability to afford health-promoting resources. Conversely, those with lower income may not have had the opportunity to pursue higher education, perpetuating a cycle of disadvantage. However, it should also be recognized that individuals with lower education but better financial resources may still meet their needs better, including access to information, healthcare, and nutrition. This interplay highlights the necessity of addressing both educational and economic disparities to develop effective interventions for sarcopenia prevention and management.

In the present study, having a low BMI (< 18.5 kg/m²) was found to be significantly more related to developing sarcopenia. This finding is consistent with those of previous studies conducted among community-dwelling older adults in Thailand [10] and Japan [23]. Additionally, in a study from China, Zhang et al. [24] reported that a high BMI served as a protective factor against the loss of skeletal muscle mass. Individuals with a low BMI are particularly prone to sarcopenia due to the lack of muscle mass and overall body weight, which directly correlates with muscle wasting. Malnutrition, commonly associated with low BMI, further exacerbates muscle loss by leading to inadequate intake of essential nutrients necessary for muscle maintenance and repair [25] which studies have demonstrated that underweight individuals are more susceptible to sarcopenia because of their limited muscle reserves and higher likelihood of nutritional deficiencies. Older adults who are malnourished often experience reduced muscle mass and strength, exacerbating sarcopenia progression. Malnutrition can result from various factors such as poor appetite, swallowing difficulties, and limited access to nutritious food. Nutritional interventions, including protein supplementation and balanced diets, have been shown to improve muscle mass and function in malnourished older adults [25].

Frailty and sarcopenia are age-related disorders that are increasingly prevalent due to global aging. This study found a correlation between frailty and sarcopenia, with a higher prevalence of sarcopenia observed in individuals with frailty compared to those without. The frequent co-occurrence of these conditions indicates a close relationship, likely due to decreased muscle strength or impaired physical function [26]. Some studies have identified sarcopenia as a significant risk factor for frailty. We recommend addressing both conditions with interventions [27,28] such as resistance training programs and multicomponent exercise interventions.

The connection between sarcopenia and falls can be attributed to several factors: sarcopenia leads to a reduction in fast muscle fibers and motoneurons, negatively impacting muscle mass, strength, and physical performance [29]. Furthermore, individuals with sarcopenia may experience endocrinopathies or malnutrition, such as vitamin D deficiency, which is linked to an increased incidence of falls and balance issues [30]. Lastly, sarcopenia is closely associated with frailty, a geriatric syndrome characterized by diminished homeostatic reserves, raising the risk of falls [31]. Consequently, sarcopenia is considered a significant risk predictor for falls in older adults.

Vitamin D deficiency was another factor significantly associated with a higher risk for sarcopenia in our population. Vitamin D is vital for muscle function and bone health [22]. Vitamin D deficiency is common among older adults and is associated with muscle weakness and heightened risk for falls and fractures. This deficiency exacerbates muscle mass and strength loss, contributing to sarcopenia. Vitamin D supplementation has been shown to improve muscle function and reduce fall incidence in deficient individuals [22].

Several chronic conditions, including stroke, knee osteoarthritis, and malignancy, were also identified as risk factors for sarcopenia in the current study. Stroke survivors are highly susceptible to sarcopenia due to prolonged immobility, muscle disuse, and neurogenic muscle atrophy [32]. The muscle weakness and reduced physical activity following a stroke accelerate muscle mass and function loss. Rehabilitation programs focusing on physical therapy and resistance training are essential in mitigating sarcopenia in patients with stroke by enhancing muscle strength and functional capacity.

Knee osteoarthritis contributes to sarcopenia through pain-induced inactivity and muscle disuse. Chronic pain limits physical activity, leading to muscle atrophy and weakness in the lower extremities. Tailored exercise programs that improve joint mobility, reduce pain, and strengthen muscles around the knee can help manage sarcopenia in these individuals [33].

Finally, our findings indicated that malignancy is related to sarcopenia. Malignancy and related treatments, such as chemotherapy and radiation, can lead to significant muscle wasting and cachexia, a condition marked by severe muscle loss. Malignancy-induced sarcopenia results from reduced food intake, metabolic alterations, and inflammation. Early nutritional support, physical activity, and interventions targeting inflammation and metabolic imbalances are crucial for managing sarcopenia in these patients.

Strengths and limitations

This study has several strengths. We used comprehensive assessment tools, including BIA, handgrip strength, and five-time chair stand tests. The diverse demographics of participants increase the generalizability of the results. Specifically, this study addresses the unique cultural and socio-economic context of Thailand by investigating factors such as income, education, living arrangements, and lifestyle behaviors. These insights help tailor public health interventions and policies to better suit the needs of older adults in Thailand. Additionally, the findings highlight the significant impact of socioeconomic factors and chronic conditions on sarcopenia.

However, the study also has several limitations. The cross-sectional design restricts the ability to determine causality between risk factors and sarcopenia. As the study was conducted in a single urban setting in Bangkok, the generalizability of the findings to other regions, particularly rural areas, may be limited. These modifications include the use of updated diagnostic criteria from the 2019 AWGS and comprehensive assessment methods such as bioelectrical impedance analysis, handgrip strength tests, and physical performance evaluations. This study adds new insights by employing the most current diagnostic standards, providing a thorough evaluation of sarcopenia, identifying additional risk factors including socioeconomic factors, nutritional status, chronic health conditions, and factors such as falling in the past year and frailty, and proposing an interaction model to explore the relationships between key risk factors and outcomes. These contributions refine and expand the existing knowledge on sarcopenia in the Bangkok population, offering a stronger foundation for targeted interventions and public health strategies. In addition, the lack of detailed socioeconomic data and control for potential confounding factors, such as genetic influences and specific dietary patterns, cannot be excluded. Finally, the assessment of physical activity may not capture long-term trends necessary to fully understand their impact on the development and progression of sarcopenia.

## Conclusions

The study identified a 19% prevalence of sarcopenia among community-dwelling older adults in Bangkok and highlighted fifteen significant risk factors, including socioeconomic and health-related elements. The multifactorial nature of sarcopenia underscores the need for comprehensive, targeted interventions that address these diverse risk factors. Future research will focus on validating an interaction model to develop personalized prevention and management strategies. Implementing cost-effective, community-based approaches and integrating sarcopenia management into primary care are essential for improving the health and quality of life of older adults.
